# Delayed ^18^F-FDG PET/CT Appearance of Urachal Adenocarcinomas

**DOI:** 10.1155/2020/3216179

**Published:** 2020-09-14

**Authors:** Lian Xu, Yiping Shi, Yining Wang, Xiang Zhou, Gan Huang, Ruohua Chen, Jianjun Liu

**Affiliations:** Department of Nuclear Medicine, Ren Ji Hospital, School of Medicine, Shanghai Jiao Tong University, Shanghai, China

## Abstract

**Background:**

Urachal carcinoma is a rare urological malignancy. Use of ^18^F-FDG PET/CT in urological oncology has developed slowly because of the urinary elimination of ^18^F-FDG. We investigated whether delayed postdiuretic ^18^F-FDG PET/CT could be used for diagnosing urachal carcinoma.

**Methods:**

This retrospective study included 6 patients who underwent delayed postdiuretic ^18^F-FDG PET/CT for the evaluation of urachal carcinoma. The delayed postdiuretic PET/CT parameters and clinical characteristics of urachal carcinoma were investigated.

**Results:**

There was no significant difference in the SUVmax between the primary tumors and the urine in the bladder before delayed diuresis (25.4 ± 19.5 vs. 42.9 ± 31.1, *P*=0.18). However, the SUVmax of the primary tumors was significantly higher than the SUVmax of urine after delayed diuresis (25.4 ± 19.5 vs. 3.5 ± 1.6, *P*=0.002). Diuretic ^18^F-FDG PET/CT was positive in all patients when compared with normal liver tissues or urine after delayed diuresis. The SUVmax, TLR, and TUR of the primary tumors were 25.4 (range: 7.2–58.9), 7.0 (range: 1.8–14.7), and 6.8 (range: 3.8–11.3), respectively. Delayed postdiuretic ^18^F-FDG PET/CT had a negative predictive value of 100% (5/5) for predicting lymph node metastasis. One patient received chemotherapy after radical resection of urachal carcinoma because ^18^F-FDG PET/CT found lung metastases, and one patient only received chemotherapy because PET/CT found peritoneal and skeletal metastases.

**Conclusions:**

Delayed postdiuretic ^18^F-FDG PET/CT is a useful tool for the preoperative evaluation of urachal carcinoma. ^18^F-FDG PET/CT may improve clinical decision making and management of urachal carcinomas.

## 1. Introduction

Urachal carcinoma is a rare urological malignancy accounting for only 0.34% of all bladder neoplasms, which extends from the bladder dome to the umbilicus [1, 2]. The most common histologic type of urachal carcinoma is adenocarcinomas. Computed tomography and magnetic resonance imaging are the commonly used imaging modalities for the diagnosis of urachal carcinoma [2, 3]. ^18^F^−^fluorodeoxyglucose (FDG) positron emission tomography (PET) has become a useful modality in cancer evaluation based on hypermetabolism observed in malignant cells [4]. However, use of ^18^F-FDG PET/CT, specifically in urological oncology, has developed relatively slowly because the radiotracer is excreted into the urine and bladder, making structures and original tumors difficult to see against the tracer background [5, 6]. It is encouraging to note that ^18^F-FDG PET/CT has been used in the diagnosis of bladder cancer through delayed postdiuretic imaging and has exhibited high sensitivity and accuracy in our recent studies [7, 8]. However, because of the rarity of urachal carcinoma, the ^18^F-FDG PET/CT literature consists only of case reports [9].

In this study, we describe the delayed ^18^F-FDG PET/CT findings in urachal adenocarcinomas, which, to our knowledge, is the first series to assess the diagnostic value of delayed ^18^F-FDG PET/CT for urachal carcinomas.

## 2. Methods

### 2.1. Patients

We retrospectively reviewed all available clinicopathological data for 6 patients with urachal carcinoma who were examined by delayed postdiuretic ^18^F-FDG PET/CT and, subsequently, histologically diagnosed by radical resection of urachal carcinoma (*n* = 5) or biopsy (*n* = 1) at the Shanghai Jiaotong University-affiliated Ren Ji Hospital from January 2017 to July 2019. Among the 6 patients, 5 patients were treated with radical resection of urachal carcinoma; the remaining one patient was treated with chemotherapy. The study was approved by the institutional review board of the Shanghai Jiaotong University-affiliated Ren Ji Hospital and was in accordance with the 2013 revision of the Declaration of Helsinki. The need for informed consent was waived in this study.

### 2.2. Delayed Postdiuretic ^18^F-FDG PET/CT Imaging

Blood glucose levels were measured and found to be less than 140 mg/dL at the time the ^18^F-FDG was administered. All patients received an intravenous 3.7 MBq/kg injection of ^18^F-FDG after fasting for at least 6 h and resting for 1 h. ^18^F-FDG PET/CT scanning from the groin to the skull base was performed using a whole-body scanner (Biograph mCT; Siemens, Erlangen, Germany) (early PET/CT imaging). After early PET/CT imaging, delayed postdiuretic PET/CT imaging was performed after 120 min of early PET/CT imaging. Patients received 20 mg of furosemide by the oral route and an extra oral intake of at least 500 mL water. Patients were asked to void frequently to reduce the urine physiological uptake of the radiotracer ^18^F-FDG. Delayed imaging covered a range of one or two bed positions centered at the location of the bladder. PET images were attenuation-corrected and anatomically correlated with low-dose CT images.

For quantitative analysis, irregular regions of interest were placed over the most intense area of ^18^F-FDG uptake on delayed postdiuretic PET/CT imaging. The SUVmax was calculated as (maximum pixel value with the decay-corrected region-of-interest activity [MBq/mL])/(injected dose [MBq]/body weight [kg]). The PET/CT images were evaluated by two experienced nuclear medicine physicians. The TLR was calculated as the SUVmax of primary tumors/SUVmax of the liver. The TUR was calculated as the SUVmax of primary tumors/SUVmax of urine (delay).

### 2.3. Statistical Analysis

The data are presented as mean ± SD. Statistically significant differences between groups were compared using the Mann–Whitney *U*-test. *P* < 0.05 was considered statistically significant. Statistical analyses were performed using SPSS, version 13.0 (SPSS Inc.).

## 3. Results

### 3.1. Patient Characteristics

The patient characteristics and delayed ^18^F-FDG PET/CT finding are listed in Table 1. There were 3 males and 3 females with an average age of 48.7 (range 35–71 years). The tumor size averaged 4.3 cm in the largest cross-sectional diameter (range 2.8–7.8 cm). Five cases appeared as solid lesions and one case appeared as mixed cystic-solid lesion. Four cases appeared as a round-like nodule or mass and two cases appeared as irregular lumps. All were adenocarcinomas. Calcifications were radiologically visible in one case. One case (Case 2) had distant metastasis in the peritoneal and bone. In our series, 5 patients (83.3%) presented with hematuria as the first clinical sign, and 1 patient (16.7%) presented with painful pelvic mass as the first clinical sign.

### 3.2. PET Appearance

**Figure 1:**
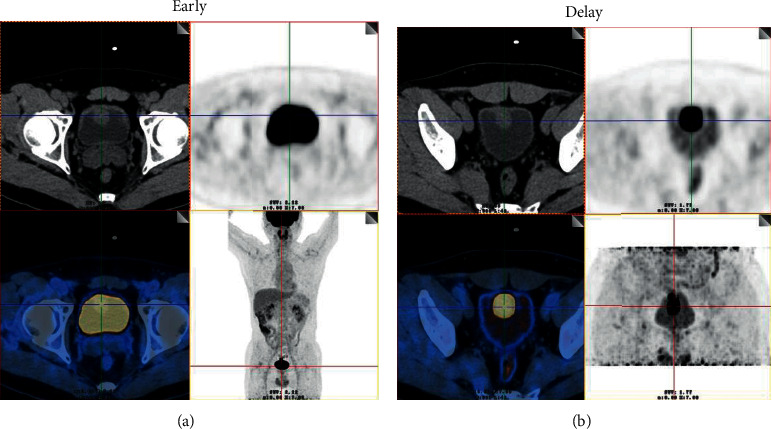
(a) Images of a 44-year-old man with urachal carcinoma (Case 3). On the ^18^F-FDG PET/CT images, axial CT shows that the space-occupying lesion was located in the bladder dome. However, the ^18^F-FDG uptake of the lesion cannot be easily detected because of urine interference in the early image (early). (b) On the image of ^18^F-FDG PET/CT in the delayed phase, the ureter is distended and the lesion can be easily visualized by axial CT and PET (SUVmax, 58.9) (delay) as the ^18^F-FDG uptake of urine in the bladder was very low (SUVmax, 5.2). The patient underwent radical resection of urachal carcinoma, and moderately differentiated adenocarcinoma was confirmed by histopathology.

The mean SUVmax of urine in the bladder of the early image was 42.9 (range 13.5–91.6), so the SUVmax of lesion cannot be easily detected due to urine interference, especially for the intraluminal portion. On the image of 18F-FDG PET/CT in the delayed phase, the bladder is distended and the SUVmax of urine was very low (mean, 3.5; range 1.8–5.2). So, the lesion can be easily visualized by axial CT and PET, particularly the intraluminal portion of the mass. Diuretic 18F-FDG PET/CT was positive in all patients when compared with normal liver tissues or urine after delayed diuresis. The SUVmax of primary tumors before diuresis was 23.6 (range 6.8–60.1).The SUVmax after diuresis, TLR, and TUR of the primary tumors were 25.4 (range 7.2–58.9), 7.0 (range 1.8–14.7), and 6.8 (range 3.8–11.3), respectively. The SUVmax of the primary tumors was significantly higher than the SUVmax of urine after delayed diuresis (25.4 ± 19.5 vs. 3.5 ± 1.6, *P*=0.002). However, there was no significant difference in the SUVmax between the primary tumors and the urine before delayed diuresis (25.4 ± 19.5 vs 42.9 ± 31.1, *P*=0.18). Representative images of a patient (Case 3) with urachal carcinoma who underwent ^18^F-FDG PET/CT scanning (early PET/CT imaging) and delayed postdiuretic PET/CT scanning are shown in Figure 1.

Of the 6 patients, 2 patients underwent ultrasound examination, 1 patient underwent computed tomography, and 3 patients underwent MRI. Though they all found the primary tumors, they did not find any metastatic lesion. Of the 6 patients, 4 patients underwent radical resection of urachal carcinoma because PET/CT found no lymph node and distant metastasis. Postoperative pathology further confirmed the ^18^F-FDG PET/CT results. One patient (Case 1) received chemotherapy after radical resection of urachal carcinoma because ^18^F-FDG PET/CT found lung metastases, though ^18^F-FDG PET/CT and postoperative pathology both found no lymph node metastasis. Thus, 18F-FDG PET/CT had a negative predictive value of 100% (5/5) for predicting lymph node metastasis. One patient (Case 2) only received chemotherapy because PET/CT found peritoneal and skeletal metastases.

## 4. Discussion

^18^F-FDG PET/CT has been widely used for diagnosis of various malignant tumors [10–12]. Unfortunately, ^18^F-FDG is not an ideal radiotracer for use in urology because of its urinary elimination [13]. In this study, the potential of delayed postdiuretic ^18^F-FDG PET/CT for diagnosing urachal carcinoma was analyzed. To the best of our knowledge, this was the first study to assess the diagnostic value of delayed ^18^F-FDG PET/CT in urachal carcinomas.

Though Yang et al. reported a case of urachal carcinoma detected by ^18^F-FDG PET/CT, it consists of only one case and delayed postdiuretic ^18^F-FDG PET/CT was untaken [9]. So, the SUVmax of urachal carcinoma cannot be easily detected due to urine interference in this case. Several investigators have considered ^18^F-FDG PET/CT of no utility in the detection of localized urological malignancies and lymph nodes [14]. The limitation of ^18^F-FDG PET/CT has been attributed to the urinary excretion of ^18^F-FDG. The pooled activity in the urinary system makes the evaluation of urinary lesions difficult. Catheterization has been proposed to reduce bladder spillover for the evaluation of the pelvic region in several studies. In addition to catheterization, our previous studies showed that washing out the excreted ^18^F-FDG could also overcome this limitation in bladder cancer [7, 8]. Delayed postdiuretic ^18^F-FDG PET/CT exhibited high sensitivity and accuracy for diagnosing bladder cancer in these studies [7, 8]. In this study, we showed that the lesion of urachal carcinomas can be easily visualized by delayed postdiuretic ^18^F-FDG PET/CT and the lesion was positive in all patients when compared with normal liver tissues or urine after delayed diuresis.

CT and MRI have been used to assess the local extent of urachal carcinomas and the presence of pelvic and visceral metastases [15]. However, they have limitations because they can detect only lymph nodes and distant metastasis that are quite enlarged. CT fails to detect lymph node involvement in up to 40–70% of patients with lymph node metastases [16]. ^18^F-FDG PET/CT was, however, more sensitive than conventional CT scan in detecting lymph node metastases based on hypermetabolism of lymph node involvement [17]. In this study, 2 patients underwent ultrasound examination, 1 patient underwent computed tomography, and 3 patients underwent MRI. Though they all found the primary tumors, they did not find any metastatic lesion. ^18^F-FDG PET/CT found no lymph node metastasis in 5 cases, and postoperative pathology further confirmed the accuracy. Thus, delayed postdiuretic 18F-FDG PET/CT had a negative predictive value of 100% (5/5) for predicting lymph node metastasis in our study. In one patient, radical resection of urachal carcinoma was not performed because PET/CT found peritoneal and skeletal metastases. Thus, delayed postdiuretic ^18^F-FDG PET/CT have the potential to improve clinical decision making and management of urachal carcinomas.

Our study was, in part, limited by its retrospective design and small sample size. Although delayed postdiuretic ^18^F-FDG PET/CT may have a good diagnostic performance for urachal carcinoma, further large and prospective studies were needed to confirm our results.

## 5. Conclusions

Our results were the first to show the diagnostic value of delayed postdiuretic ^18^F-FDG PET/CT in the urachal carcinomas. These results may advance the development of noninvasive strategies to improve clinical decision making and management of urachal carcinomas. Further larger and prospective studies that include more clinical samples are needed to confirm the value and efficacy of delayed postdiuretic ^18^F-FDG PET/CT in urachal carcinomas.

## Figures and Tables

**Table 1:** Patient characteristics and results of PET and CT scan.

Case	Gender	Age	SUVmax of the lesion	SUVmax of urine (early)	SUVmax of urine (delay)	SUVmax of the liver	History	Conventional Imaging methods	Size (mm)	Morphology and density	Metastasis	Treatment
1	Female	35	18.9	13.5	2.6	2.3	Hematuria	Ultrasound	40	Round-like mass, solid	Lung	Surgery and chemotherapy
2	Male	52	38.2	91.6	5.1	3.6	Hematuria	Ultrasound	78	Irregular lumps, solid	Peritoneal, skeletal	Chemotherapy
3	Male	44	58.9	51	5.2	4	Hematuria	MR	30	Tubercle like, solid	None	Surgery
4	Female	71	16.8	63.2	4.4	4.2	Pelvic mass	MR	50	Irregular lumps, cystic-solid	None	Surgery
5	Male	52	12.3	19.4	1.8	4.2	Hematuria	CT	28	Round tubercle like, solid	None	Surgery
6	Female	38	7.2	18.9	1.8	4	Hematuria	MR	30	Tubercle like, solid	None	Surgery

## Data Availability

The data used to support the findings of this study are available from the corresponding author upon request.

## Abbreviations

^18^F-FDG PET: ^18^F^−^fluorodeoxyglucose positron emission tomography
SUVmax: The maximum of standardized uptake value.

## References

[1] Brick S. H., Friedman A. C., Pollack H. M. (1988). Urachal carcinoma: CT findings. *Radiology*.

[2] Fishman C. M., Woodward P. J., Wagner B. J. (2005). Computed tomographic appearance of urachal adenocarcinomas: review of 25 cases. *European Radiology*.

[3] Monteiro V., Cunha T. M. (2012). Urachal carcinoma: imaging findings. *Acta Radiologica Short Reports*.

[4] Plathow C., Weber W. A. (2008). Tumor cell metabolism imaging. *Journal of Nuclear Medicine*.

[5] Kitajima K., Yamamoto S., Fukushima K., Minamimoto R., Kamai T., Jadvar H. (2016). Update on advances in molecular PET in urological oncology. *Japanese Journal of Radiology*.

[6] Mafeld S., Vasdev N., Patel A. (2015). Evolving role of positron emission tomography (PET) in urological malignancy. *BJU International*.

[7] Ali R., Zhou X., Liu J., Huang G. (2019). Relationship between the expression of PD-1/PD-L1 and 18F-FDG uptake in bladder cancer. *European Journal of Nuclear Medicine and Molecular Imaging*.

[8] Yan H., Zhou X., Wang X. (2019). Delayed (18) F FDG PET/CT imaging in the assessment of residual tumors after transurethral resection of bladder cancer. *Radiology*.

[9] Yang G., Wang Z., Liu S., Wu F., Li D. (2017). A rare case of urachal carcinoma metastatic to thoracic vertebra detected by FDG PET/CT. *Clinical Nuclear Medicine*.

[10] Zhou X., Chen R., Xie W., Ni Y., Liu J., Huang G. (2014). Relationship between 18F-FDG accumulation and lactate dehydrogenase A expression in lung adenocarcinomas. *Journal of Nuclear Medicine*.

[11] Chen R., Wang Y., Zhou X., Huang G., Liu J. (2018). Preoperative PET/CT (18) F-FDG standardized uptake by lymph nodes as a significant prognostic factor in patients with colorectal cancer. *Contrast Media & Molecular Imaging*.

[12] Wu C., Chen R., Zhou X., Xia Q., Liu J. (2020). Preoperative evaluation of residual tumor in patients with endometrial carcinoma by using 18F-FDG PET/CT. *Journal of Cancer*.

[13] Razik A., Das C. J., Sharma S. (2018). PET-CT and PET-MR in urological cancers other than prostate cancer: an update on state of the art. *Indian Journal of Urology*.

[14] Schöder H., Larson S. M. (2004). Positron emission tomography for prostate, bladder, and renal cancer. *Seminars in Nuclear Medicine*.

[15] Voges G. E., Tauschke E., Stöckle M., Alken P., Hohenfellner R. (1989). Computerized tomography: an unreliable method for accurate staging of bladder tumors in patients who are candidates for radical cystectomy. *Journal of Urology*.

[16] Paik M. L., Scolieri M. J., Brown S. L., Spirnak J. P., Resnick M. I. (2000). Limitations of computerized tomography in staging invasive bladder cancer before radical cystectomy. *The Journal of Urology*.

[17] Nayak B., Dogra P. N., Naswa N., Kumar R. (2013). Diuretic 18F-FDG PET/CT imaging for detection and locoregional staging of urinary bladder cancer: prospective evaluation of a novel technique. *European Journal of Nuclear Medicine and Molecular Imaging*.

